# The Meaning of “Clean” in Anti-doping Education and Decision Making: Moving Toward Integrity and Conceptual Clarity

**DOI:** 10.3389/fspor.2022.869704

**Published:** 2022-05-13

**Authors:** Andrea Petróczi, Ian D. Boardley

**Affiliations:** ^1^Department of Applied and Human Sciences, Kingston University London, Kingston upon Thames, United Kingdom; ^2^Katholieke Universiteit Leuven, Leuven, Belgium; ^3^School of Sport, Exercise, and Rehabilitation Sciences, University of Birmingham, Birmingham, United Kingdom

**Keywords:** anti-doping, integrity, values based education, decision making, international standard, education, spirit of sport, sense-making

## Abstract

With the World Anti-Doping Agency's International Standard for Education (ISE) coming into effect in 2021, the clean-sport movement is at a pivotal stage. Through this conceptual paper we juxtapose the sector-wide anti-doping education as set out in the ISE on the decision-making process at the individual level. We discuss three critical issues for the clean-sport movement. First, we make the case for doping being a “wicked” problem and outline the possible implications of this for prevention and detection. Second, we consider why we need to address regulative, normative, and cognitive components of clean sport if we are to maximize its legitimacy. Third, we critically expose the fluidity with which clean sport is defined, and the implications of defining clean sport in substance- vs. rule-based terms, which, respectively, lead to theorizing clean sport as “drug-free” vs. “cheating-free” sport. Finally, we consider the role and key components of anti-doping education and how the relevance of certain components may be dependent on the way clean sport is defined. Conceptualizing doping as a sport integrity issue, we move away from the archaic and delimiting view of clean sport as drug-free sport and conclude with recommendations on how to reconcile values-based education, awareness raising, information provision and anti-doping education within the broader scope of integrity, to support informed decision making and personal agency. To connect anti-doping education to individual-level decision making, we recommend a staggered approach in which specific education content is linked to different influences in the decision-making process, to different stages of athlete development, and to different educational goals. Emphasizing and encouraging sensemaking in anti-doping decision making offers a pragmatic approach for anti-doping education. Conceptual clarity and precise mapping of the educational goal, content, and delivery is vital for valid and meaningful evaluation of the effectiveness of anti-doping education.

## Introduction

Young people are attracted to sport for a variety of reasons including quests for excitement, participation, health, competition, acknowledgment, prestige, and profit. What differentiates elite athletes from their non-elite counterparts is their ability/talent, their desire to compare and contest this against other elites, and thus an infinite drive and need for constant performance enhancement. Because of this, specific rules have been put in place to ensure performances and achievements are comparable. For example, we have weight categories in sports where mass has a large impact on performance, or disability classification in para-sports to try to ensure fairness. Equipment and apparel (e.g., sharkskin swimsuits in swimming; running shoes with carbon plates) in sport are often regulated, and new equipment is sometimes withheld until everyone has had the opportunity to train with it (e.g., clap skates in speed skating). Any breaking of such rules constitutes cheating and is therefore sanctioned within sports.

The global anti-doping movement was formed for similar reasons, and to help determine the boundaries between prohibited and non-prohibited forms of performance enhancement. Since its formation in 1999, the World Anti-Doping Agency (WADA) has led this movement globally, by determining which substances and methods are deemed illicit based upon their impact on health, performance and/or violating the spirit of sport. The anti-doping movement has faced many challenges in the 20 years since WADA was formed, and it is important that we continue to improve anti-doping efforts. To reduce and deter doping use, a holistic approach that addresses requisite cultural, economic, and social changes with input from all relevant research disciplines, stakeholders, sponsors, and industry partners is needed (Pitsiladis et al., 2019). Signatories of Pitsiladis's et al. (2019) declaration argue this holistic and concerted effort is required because:

“*[D]oping, and cheating in general, threatens to eliminate the essence of sport and the embodiment of the Olympic ethos and spirit. Doping practices, and the persistent suspicion of them, casts doubt on athletic achievements at the limits of human capabilities. It is clear that the public at large desire clean and fair sport and that athletes want to compete in a clean sport environment providing strong legitimacy to anti-doping efforts*.” (p. 448)

Holistic approaches call for careful consideration of how education can and should support anti-doping efforts in the most meaningful way. In this paper we look at anti-doping education, its approaches, goals, and potential evaluation through the athlete's point of view. To make education practically relevant and meaningful, we connect the current policy-driven anti-doping education, as set out in the International Standard for Education (ISE; WADA, 2021a), to individual level decision-making processes. However, to be able to do so, we first seek to understand the context in which individual decisions take place. Specifically, we explore the reasons why making sport ‘clean' is difficult, how anti-doping likely appears from the athletes' point of view, and consider the challenges and potential solutions for anti-doping education in this context.

## Doping as a Wicked Problem

Previously anti-doping scholars have described doping as a wicked problem, drawing comparisons between doping and problems that have been resistant to resolution through social policy (e.g., Kazlauskas, 2007; Pielke, 2016; Schultz, 2019; Viret, 2020; van Bottenburg et al., 2020). Wicked problems are those where a solution is difficult or impossible to find due to deficient information and conflicting and shifting requirements that are frequently hard to recognize and/or not evident until an initial attempt is made to solve the issue. Rittel and Webber (1973) identified 10 characteristics of wicked problems. As we have done in Table 1, it is possible to apply Rittel and Webber's (1973) ten characteristics to doping, providing support for the contention that doping indeed represents a wicked problem.

**Table 1:** Characterizing doping as a wicked problem.

**General attribute**	**Attribute manifesting in doping**
1. There is no definite formulation: Wicked problems are tough to describe. To describe a wicked problem, one must develop an exhaustive inventory of ways to solve the problem before the problem is even identified. The process of describing the problem, and formulation of solutions are identical and simultaneous, not sequential like in tame problems.	Doping use and the doping problem are distinct issues. Doping use is concerning for stakeholders. It becomes a problem because rules are set to address the concerns and having formal rules that necessitate enforcement.
2. There is no stopping rule: With tame problems, it is clear to everyone when the problem is solved (e.g., an airplane is built, a data management system is implemented) with a definite handover or launch moment. With wicked problems, this is not the case because the process of describing the problem and formulation of a solution is simultaneous. There is no set of criteria against which the problem solver can judge the solution and determine whether the optional solution was reached. Stopping problem solving in wicked problems are arbitrary decisions and usually linked to unrelated factors such as running out of time, resources, patience, enthusiasm or pressured by some external deadline.	Total absence of drugs in sport is an impossible situation as: (1) there is no way to quantitatively define what is “good enough” and (2) stakeholders are likely to disagree on what is “good enough.” Effective anti-doping is relative in terms of being defined by making improvement—anchored to a status quo at a given time point—rather than an absolute target independent of the status quo.
3. Solutions are not true or false but good or bad: Because there is no set of objective criteria to which a solution can be compared, there is no way of judging whether the solution is the correct one. Part of the reason for not having an objective set of criteria is the nature of the problem and the different conceptualization of the problem/solution by different stakeholders. There is no independent qualified person or organization who can judge the solution, the problem solvers are the judges.	History suggests the existing anti-doping strategies, namely prevention *via* education and control *via* detection and sanctioning, are not capable of eradicating the doping problem. Drastic suggestions such as eradicating the doping problem by removing doping control would not be an acceptable solution for stakeholders. Furthermore, there is no agreed objective criteria against the success of anti-doping efforts can be judged. Naturally, the prevalence of doping could be one such objective criterion, but it requires two conditions to be met: (1) an agreement on what success is—whether it is zero rate of doping (complete eradication) or a sufficient control and suppression (but non-zero prevalence); and (2) the way to measure prevalence of doping in a reliable and robust manner.
4. There is no immediate and no ultimate test of a solution: In tame problems, testing of a solution is pre-determined and under control, with a clear pass or fail outcome. In wicked problems, implementing the solution immediately generates a plethora of consequences over an undefined period—which now also need solutions. To fully anticipate the potential consequences in advance is impossible. Often the consequence is not even obvious until after the solution is put in place.	Controlling the use of certain substances in sport called for developing tests for these substances, which led to implementing sampling and testing protocols in- and out-of-competition. The latter then called for having information on athletes' whereabouts so they can be tested unannounced.
5. Every solution is a one-shot operation: Because there is no opportunity to learn and define, every attempt counts. In addition to the wasted resources, failed attempts do not get forgotten. They leave a legacy that the next solution must consider. Every attempt creates a new situation that calls for a solution, and every attempt to correct previously failed attempts creates a host of new wicked problems. There is no way to return to the previous status quo and try a different solution.	In sport, defining moments in athletes' careers are linked to major sport events, which are one-off operations. Athletes rely on the organizers of such events, their respective sport federations, and global anti-doping to ensure a safe and fair competitive environment. Any rules implemented for these events impacts athletes' performance, competitive outcomes, and chances for a desired result. For athletes and doping controllers, every attempt counts.
6. There is no definite set of potential solutions: Because wicked problems do not have a well-described set of potential solutions, it is impossible to show that all possible solutions were considered. It is also possible that no solution is found because of the inconsistencies between definitions of the problems by different stakeholders.	The doping problem manifests differently for different groups (e.g., athletes, coaches, NADOs, IFs, WADA, IOC), therefore different—and often conflicting—solutions co-exist. For example, a national Anti-doping organization's take on tackling doping in sport at all levels finds a mission conflict between public health and harm-reduction approaches suitable for fitness sport and anti-doping rule compliance at the elite competitive level. One solution cannot serve the full spectrum. Furthermore, the diversity across the 11 ADRV's suggests every individual doping problem is likely to be unique, which makes finding a single solution impossible.
7. Every problem is unique means that there is no benefit from previous experiences. Wicked problems may have similarities but there is at least one particular aspect that overrides the similarities. The consequence of every problem being unique is that no general guidance or rules can be developed which are applicable to a ‘group’ (or classes) of problems. There is no learning process in place. Attempts to use previously tested and familiar tools can make the problem worse, not better.	The doping problem as recognized in the early 20th century was unique. Problem solvers intuitively reached for a similar problem to take a solution framework from. This phenomenon is recognised as Maslow's rule of instrument which states that if the only tool you have is a hammer, it's tempting to treat everything as it were a nail. This is a cognitive bias that involves an over-reliance on a familiar tool and favours familiar instruments, but for doping control it has made the situation more complicated, not simpler. Grown out from health concerns, the doping problem was first framed as a ‘drug problem’, which was later justified on ethical grounds as being against the ‘Spirit of Sport’. This led to the much debated ‘two out of three’ criteria for prohibiting a substance.
8. Every wicked problem can be a symptom of another problem, and this only becomes apparent when one solution is implemented: In complex but tame problems, we define a problem as a gap between the current and the desired state and attempt to solve it by removing the reason for the gap. In wicked problems, removal of the cause does not solve the problem, it only makes it apparent that what was perceived as a problem at the local level is actually a symptom of a problem at a higher level. The higher one goes, the more complex the problem becomes, which makes it even more difficult to define the problem and find a solution.	The complexity of doping means isolated problems can soon create many problems to resolve. Doping control mechanisms are continuously expanded to address emerging issues. However, the needs for new solutions are not limited to new substances and better detection methods but also include needs generated by the previously implemented doping controls (e.g., prohibition of a substance and ways of detection; out of competition testing and the whereabouts system).
9. The gap between the current and the desired states presenting the wicked problems can be explained in different ways. The choice of explanation determines the nature of a solution, as well as the evaluation of the solution. Localized definitions are likely to capture only a segment of the problem. In contrast, a more realistic, global, holistic view is too complex to describe.	Depending on how doping and clean sport is conceptualized, doping can be seen as a health problem (i.e., short- and long-term consequences), a drug problem (i.e., substance misuse or abuse) or a deviance issue (i.e., rule breaking), or a symptom of a bigger unresolved issue around the ethics and governance of human enhancement. Attempted solutions tend to follow the same logic and lead to a toolbox of public health (e.g., awareness raising), law (testing and sanctioning), or moral education.
10. The planner has no right to be wrong: In science where a solution (i.e., explanation for a problem) is formulated as a hypothesis and subjected to repeated testing. If at a later point the solution is refuted, it is seen as advancing knowledge, and a normal part of scientific discovery. Solvers of wicked problems do not seek the ultimate truth but try to make the world better. Because they impact on people's lives, their mistakes are not forgotten. They are liable for the consequences of the actions they generate.	Even though doping is a complex issue to address, policymakers still have a responsibility to think of the consequences of their actions as they are accountable to many stakeholders. Attempts and failed attempts affect athletes' lives, and the effect is irreversible. For example, athletes losing Olympic medals for overzealous implementation, lives affected by doping accusations, or a clean athlete losing out on a podium moment to a doping athlete. The impacts are far-reaching and irreversible.

Recognizing that problems are not categorically wicked or not but present on a continuum (Alford and Head, 2017), the set of 10 characteristics have been expanded to categorize “super wicked” problems by adding four additional characteristics: time is running out, lack of central authority or only a weak central authority to manage the problem, the same actors causing the problem are entrusted to solve it, and irrational discounting that pushes responses into the future (Levin et al., 2012). Turnbull and Hoppe (2019) also operationalized “wicked problems” as a continuum based on the degree of “problematicity” or “structuredness” of problems and substituted the “wicked” label with “political distance” to describe how differences in values, economic and political interests, institutional authority, and diversity of implementation practices lead to a degree of distance between stakeholders. The political distance in anti-doping is exemplified in the discontent by athletes over politically motivated decisions after a nationwide ban (e.g., allowing Russian athletes to participate in the Olympics), the increasingly vocal interest groups (e.g., Global Athlete), and the emergence of alternative or localized clean sport initiatives (e.g., the Clean Sport Collective; The Clean Protocol; QUARTZ).

Taking the degree of “wickedness” or “problematicity” of the doping problem into account is critical on multiple grounds. Firstly, aspirations and expectations for a solution for doping, especially when stated as a desired permanent state such as eradication of doping from sport, must be carefully considered. Secondly, this wickedness impacts legitimacy perceptions (Woolway et al., 2020). If there is a gap between organizational mission statement, declared values, and people's everyday experiences with anti-doping (Gleaves and Christiansen, 2019; Woolway et al., 2020), it affects people's perceptions about the anti-doping policies, and in turn affects support for such policies (Petróczi, 2021; Shelley et al., 2021; Barkoukis et al., 2022). Thirdly, if we accept that doping represents a wicked problem, then this should have implications for how we address the doping problem through education. As wicked problems are situated and dynamic, any attempt to “fix” the issue by finding a single solution is not only doomed to failure but can potentially make things worse. To explain, responses to wicked problems should not be considered a matter of exploring, finding, and deciding upon the right course of action, but instead planning to constantly seek collective and distributed responses to Rittel and Webber's (1973) question for any wicked problem, “Is this the right thing to do?”. Taming a wicked problem should not be about finding a conclusive truth but instead trying to constantly improve our response to the identified need. Thus, in line with Jordan et al. (2014) our aim should be to understand and utilize wicked problems as frameworks for responding to problems within anti-doping education.

One key characteristic of wicked problems is that they are ill-defined, which makes developing effective solutions to address them very challenging. If the problem is ill-defined, it not only makes identifying the aims of anti-doping efforts difficult, but also renders measuring effectiveness problematic too. Bore and Wright (2009) examined teacher preparation as a wicked problem, focusing specifically on policy formation, implementation, and service provision. In their analysis they warned of how silo mentality models (e.g., academic, professional, political) depend upon individual nominal languages and practices that present a barrier for effective communication between members of different silos. This issue has apparent relevance to anti-doping, where different groups (e.g., WADA, NADOs, and athletes) have different perceptions of the problem and therefore solutions to it.

Framing anti-doping as a wicked problem should allow the exploration of alternative approaches to those adopted to date (Bore and Wright, 2009; Barrett, 2012; Southgate et al., 2013; Peters, 2017). Doing so suggests decision makers require more tentative and contextually driven responses and actions. More specifically, it would indicate the need for greater negotiation and meaning making to facilitate continual reinterpreting (i.e., receptivity to shifts in understanding), resolution formation (i.e., receptivity to shifts in actions), and resolving (i.e., receptivity to open-endedness not closure). Approaches that appreciate and embrace the complexity of wicked problems shun problem solving that seeks to identify and adopt single-pronged solutions. Jordan et al. (2014) put forward three approaches that have apparent applicability to addressing doping as a wicked problem: (a) promoting careful observation and continuous curiosity; (b) increasing conversations with diverse stakeholders; and (c) engaging in collective and distributed sense-making. By approaching doping as a wicked problem, we could help address acknowledged issues with the legitimacy of anti-doping and clean sport.

## Legitimacy of Anti-Doping and Clean Sport

Legitimacy is the fundamental constituent of voluntary compliance with the law or with specific rules. The legitimacy of anti-doping is built on the drive to preserve the integrity and spirit of sport to which doping is seen as being “fundamentally contrary.” Rules and organisations are in place to establish system-level legitimacy of anti-doping (Read et al., 2021). However, because preventive efforts target individual athletes and individuals in the athlete entourage, it is vital to understand how legitimacy of anti-doping is perceived by those who are directly affected. Legitimacy of anti-doping as perceived by athletes is determined by the combination of shared ideals about clean sport, which justifies the existence of anti-doping, and its procedural fairness and effectiveness (Woolway et al., 2020).

To understand sources of legitimacy for clean sport and behavioral reasoning around compliance with anti-doping, we can examine competitive sport as a social institution. Previous theorizing has focused on regulative, normative, and cognitive systems as three key facets of institutions (Scott, 1995). Although distinct, these three facets do not exist independently of one another, and instead reflect different levels or views of an institution. For example, economists and legal scholars may see institutions as judicial systems, sociologists might view them as normative systems, whereas psychologists may emphasize the role of individuals and their thought processes (Scott, 1981, 1995; Meyer and Scott, 1983). Rather than any one of these three viewpoints being accurate, the most effective way to understand legitimacy likely entails considering the contributions of all three.

Regulative, normative, and cognitive systems within anti-doping are comprised of distinct components that differ in the philosophy that underpins the operation of these components to prevent doping. The regulative system consists of policies, rules, and regulations, and the legitimacy of this system resides in the legality of the institution (i.e., WADA) that establishes and executes the policies, rules, and regulations. The assumption underpinning the operation of this system is that athletes and those who support them will be compliant to avoid being sanctioned for contravening one or more of the anti-doping rules. The World Anti-Doping Code (WADA, 2021b) represents the regulatory basis of anti-doping. The 2021 code describes how anti-doping rule violations consist not just of use or possession of illicit substances/methods, but also considers enablers and facilitators of doping. In total, there are now 11 ADRVs in the 2021 Code, including possession, assistance, trafficking, and non-compliance on whereabouts or during doping control sample collection. Only two of the 11 relate directly to the (attempted) use of prohibited substances/ methods (WADA, 2021b,c). In contrast, the normative system represents the shared norms, habits, and local practices relevant to anti-doping. Collective moral and ethical standards within sport—rather than formalized rules and prohibition—form the basis of legitimacy for the normative system in anti-doping. Within this system, athletes and their support network are proposed to comply with anti-doping because they perceive a collective belief within sport (e.g., spirit of sport) that suggests this is the right thing to do. Although collective in nature, the degree to which such beliefs are perceived to be salient is likely to be far more localized than the normative standards which are imposed at the global level. Finally, the cognitive system consists of an individual's identity, beliefs, and assumptions. Legitimacy within this system lies with the cultural systems and micro-environments that surround athletes. Here, athletes willingly avoid doping because being clean is part of their sense of self (Petróczi et al., 2021). The influence of the social environment on the cognitive system is proposed to be even more localized than that for the normative system.

**Figure 1:**
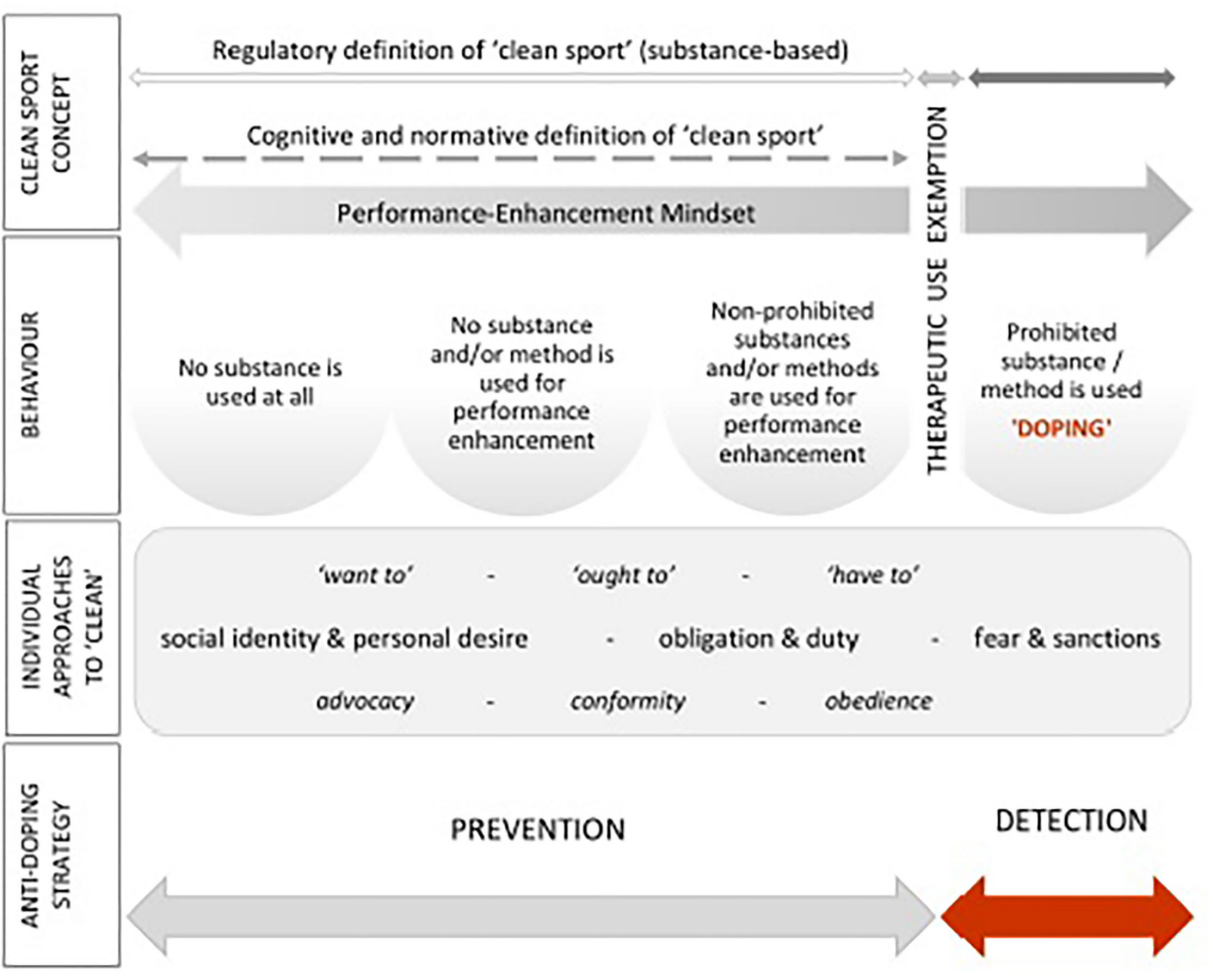
Regulatory, normative, and social cognitive context of performance enhancement and dean sport behavior.

In Figure 1 we draw a conceptual map of the key constituents of the anti-doping system. In contrast to static models that focus on stepping across the regulative barrier such as the Sport Drug Control Model (Donovan et al., 2002), Prototype Willingness Model (Whitaker et al., 2014), or the integrated model of doping use (Lazuras et al., 2015), this conceptualization builds on dynamic and/or situated models (e.g., Johnson, 2011; Petróczi, 2013; Hauw, 2013). The proposed model is situated to allow the positioning of any relevant behavior (e.g., complete abstinence from substance use; supplement use; doping use) across regulative (i.e., institutional), normative (i.e., social), and cognitive (i.e., personal) regions. It is also dynamic to account for the proposed instability of any individual's position, thus allowing them to shift across the athletic lifecycle. The model does not, however, suggest that athletes naturally progress from one position to another as they move through their career. Instead, they can adopt any position on the continuum at any point in their career, dependent upon the confluence of the multitude of competing factors that will determine this position.

A range of bases are proposed by different groups to establish the rationale for the need for anti-doping, as well as its invasive and challenging procedures. One example is the rationale forwarded by philosophers and ethicists, who propose anti-doping is warranted because the public want fair competition (e.g., Bloodworth and McNamee, 2017; Loland and McNamee, 2019), or healthy sport (e.g., Murray, 2016). Alternatively, the media and sponsors desire a clean and true image for sport because this makes it more marketable as a product (e.g., Kreft, 2011; Frenger et al., 2013). At the same time, psychologists and educators argue the need for anti-doping because athletes themselves call for a clean-sport environment to compete in (e.g., Shelley et al., 2021; Petróczi et al., 2021).

Dissenting voices question the procedural legitimacy of anti-doping on the basis of definition and detection (e.g., Pitsch, 2009, 2011; Pielke and Boye, 2019; Nissen-Meyer et al., 2022), invasion of privacy (e.g., Malloy and Zakus, 2002; Elbe and Overbye, 2014), normative foundation (e.g., Heuberger et al., 2021), legal position and the need for evidentiary evidence (e.g., Viret, 2018), and protection of vulnerable athletes (e.g., Kleiderman et al., 2020). Critiques based on whether athletes are sufficiently prepared and educated for their Code-mandated responsibilities consider the effectiveness of anti-doping education to prevent accidental as well as deliberate and motivated rule violations (e.g., Woolf, 2020; Qvarfordt et al., 2021). Taking a controversial position of allowing doping in sport, some authors argue that in comparison to an ill-fitted, ineffective and/or struggling anti-doping system, legalising doping at least would bring several benefits (e.g., Savulescu et al., 2004; Kayser et al., 2005; Henning et al., 2021). These include allowing for appropriate safety measures and medical control, bridging gaps between amateur, fitness, and competitive sport, and avoiding the awkward demarcation between sport and non-sport contexts for psychoactive drugs.

Despite different stakeholder groups justifying the need for anti-doping on different grounds, the suitability of its regulatory system should be determined not just by its approval (i.e., doing what is right), but also by its effectiveness and fairness (i.e., doing it in the right way) (Tyler, 2006; Woolway et al., 2020). Thus, those looking to provide direction for the anti-doping movement should aim to address approval, effectiveness, and fairness and involve^1^ the full range of stakeholders when doing so to ensure the legitimacy of anti-doping for all stakeholder groups.

Empirical evidence exists for the positive impact of anti-doping education on perceived legitimacy of anti-doping, which in turn positively impacts on athletes' compliance with anti-doping, as well as advocating for anti-doping (Barkoukis et al., 2022). However, we feel compelled to draw attention to the conceptual difference between clean sport behaviour and anti-doping code compliance based on two fundamental grounds. One is the definition of “clean sport” and whether it can be limited to anti-doping, and the other is the fact that clean sport behaviour and anti-doping code compliance are underpinned and driven by different motives. Anti-doping education strategies and evaluation plans must be mindful of this fundamental difference to avoid scenarios where information-based education for code compliance, a valid education goal on its own right, expects to foster clean sport behaviour. Organisations with responsibility for anti-doping education should resist limiting values-based education to the “spirit of sport” condition. Normative and relevant today as they might (e.g., Loland and McNamee, 2019), these abstract values can justify anti-doping at the system level, but they lack direct relevance for individual-level decision making about anti-doping code compliance or clean sport behaviour. Rather, they are operationalised through situational meaning and meaning-making processes (Park, 2010).

## Definition of Clean Sport

If we are to try to uphold and promote clean sport through effective education, it is important we have a clear and consistent definition of what clean sport is. However, to date its definition has been either opaque, inconsistent, or both. For instance, whilst some acknowledge that clean sport goes beyond the absence of doping, many still equate clean sport with drug-free sport. There are many other threats to the integrity of sport that do not involve doping (Petróczi, 2021). This is clear in the Olympia Declaration, which states “doping, and cheating in general, threatens to eliminate the essence of sport” (Pitsiladis et al., 2019, p. 448). As such, it is important any definition of clean sport acknowledges and clarifies the representation of these other integrity issues alongside doping. To convey our thinking on this, below we outline two extreme and impractical operationalizations of the term and identify the issues we see with them, before proceeding to describe alternative and more workable uses of the term.

One extreme interpretation of clean sport is that it represents “drug-free” sport. Such a definition soon falls down when one recognizes athletes with no intention of using prohibited substances or methods can legitimately enhance their performance with non-prohibited drugs (e.g., caffeine) and treat illnesses with both non-prohibited and otherwise prohibited medications if exemptions are granted. Given this, clean sport cannot be defined in these terms and should not be interpreted as a proxy for “drug-free” sport as it represents a very extreme position that only a very small percentage of athletes are likely to adopt. Another extreme use of the term is to indicate not using exogenous means of performance enhancement. However, most would acknowledge that this Corinthian view of sport is outdated and ignorant of the quite widespread use of diet manipulation, functional foods, licit supplementation, and licit training aids to enhance performance (Knapik et al., 2016). If promotion of clean sport is to be widely accepted by athletes, its representation needs to acknowledge performance-enhancement *per se* is not an undesirable behavior, only when it is achieved *via* prohibited means (Petróczi et al., 2017).

Anti-doping currently aligns clean sport with a substance and method-based definition in which clean sport represents not using substances or methods that are prohibited in sport. This definition benefits from its alignment with anti-doping control and testing, which is designed to catch and sanction athletes who have ingested a prohibited substance or used a prohibited method. A major limitation of this interpretation though is the focusing of deterrence and education on ensuring athletes comply with the WADA code rather than on developing athletes' clean-sport values and critical thinking abilities. Hence, clean-sport education is largely limited to telling athletes what they can and cannot do and the consequences if they—intentionally or inadvertently—perpetrate an ADRV. Because values of sport are not attached to specific drugs or drug groups, only to their position relating to the actual, in force, Prohibited List of WADA (2021a), values-based justification of anti-doping implicitly introduces cheating and rule breaking into the picture.

An alternative definition of clean sport would be to adopt a rule-based definition that conceptualizes clean sport as cheating-free sport. Here, clean sport is defined in terms of rule compliance, whereby clean athletes respect the rules and if all athletes compete in this way victory and performance is solely determined by natural abilities and effort. Under this definition, clean sport encompasses all forms of cheating, with doping representing just one form of rule infringement. This definition is consistent with the beliefs and actions of some elite athletes, as shown through focus-group interviews with elite athletes from five European nations (Petróczi et al., 2021). A key theme identified in these focus groups was the belief that clean sport is not merely drug-free sport but cheating-free sport. There was an implicit agreement amongst many athletes that doping is unacceptable because it breaks the rules of sport, not because of the drugs *per se*. One major advantage of a rule-based definition is that it is consistent with a contemporary view of doping as a sport integrity issue. Adopting a definition whereby doping is framed as a form of cheating, being a clean athlete would mean not engaging in any form of cheating. Avoidance of doping would sit alongside other cheating behaviors such as faking injury, manipulating performance to avoid a certain opponent in subsequent rounds, and classification fraud in disabled sport. In adopting this definition, we would assume athletes do not differentiate doping from other forms of cheating, which to date has not been tested through empirical examination except in one study by Šukys et al. (2019) which—in line with Petróczi (2021)—largely supports the notion that doping is considered one form of cheating among the array of other infringements. One major challenge to this approach is finding ways to differentiate between inadvertent doping and deliberate goal-oriented ADRVs. Often only the latter is conceptualised as “doping” in research, but both advertent and inadvertent “doping” fall under ADRVs and are sanctioned according to the WADA Code (WADA, 2021a). This would have to be addressed before this definition could be adopted.

## Role of Integrity in Clean Sport

### Operating Within the “Clean” Zone

The term “integrity” is used frequently in the anti-doping literature, boasting sizeable literature presence (e.g., Treagus et al., 2011; Agnew et al., 2017). At the same time, the concept still lacks a robust operational definition and taxonomy that differentiates between the integrity of sport, the integrity of a specific sport or anti-doping organisation, the integrity of anti-doping policies and procedures, or the integrity of a person involved in anti-doping in some capacity (Cléret et al., 2015). Gardiner and colleagues argue against the casual use of this term because such use obfuscates the reality that these terms (i.e., integrity and related concepts such as corruption, cheating) are complex, imprecise, and contested concepts” (2016, p11).

*Ad hoc* definitions of “integrity” in the sport context are variably linked to a range of sport values and moral principles, and investigated through concepts such as sportspersonship, fair play, respect, positive personal values of responsibility, compassion for the other, and honesty in adhering to rules (Treagus et al., 2011), or socially constructed through norms for expected behaviour dictated by rules (Ordway and Opie, 2016) and/or customs (Loyens et al., 2021). Presenting an overview of philosophical and psychological perspectives of “integrity”, Gardiner et al. (2017) differentiate between moral and behavioural integrity and personal and organisational integrity, flagging the difference between integrity as a quality of an entity (e.g., organisations, policies), and individuals who comprise or are responsible for these entities. The difference between actual and perceived integrity of an entity must also be flagged for attention, with the latter reflecting not on the entity, but the person making the judgement about the (perceived) integrity of some rule, process, organisation, or person.

Relevant to the focus of our argument, is Gardiner et al.'s (2017) concept of “personal integrity,” which is the amalgamation of the philosophical approach through ‘commitment’ and the psychological approach of “being true to oneself” through sense-making and the decision-making process itself. Personal integrity manifests in behavioural choices, resulting in consequences stemming from individual decisions. In this sense, integrity is one's ideal self, and involves: (a) self-awareness of one's value systems, (b) having an honest, open, and critical review of context, aspirations, and the limitations of personal goals and authenticity (Cottingham, 2010; Robinson, 2016), and (c) recognition and management of contradictions and inconsistencies though prioritising and re-prioritising values and goals (Parks-Leduc and Guay, 2009).

## Operating Within the “Clean Zone”

Whilst it may situate doping in a broader range of integrity issues, adopting a rule-based definition does not inherently address the issue of a focus on telling athletes what they can and cannot do and the consequences of rule non-compliance. This issue is particularly apparent when one considers the wide range of contrasting behaviors that would all be categorized as “clean” if one merely applied the regulatory definition of clean sport (see Figure 1). Importantly, the width of this acceptable zone of behaviors can vary markedly from one athlete to another (Fincoeur et al., 2020; Petróczi et al., 2021). For some, it is fine to operate toward the right-hand side of this zone within what is often referred to as the “gray zone,” whereby behavior is close to but does not breach the “hard line” of prohibition. At this point, whilst certain practices may not be against the rules, at times they likely violate the spirit of sport (e.g., off-label use of medications). Others, however, stay well clear of such behaviors, operating instead exclusively toward the left-hand side of this zone. When operating in this “clean” zone, personal boundaries guide actions rather than the regulatory framework. The issue here though, is that the wide variety of approaches to performance enhancement that athletes adopt vary considerably in terms of the performance advantage they convey, with some behaviors toward the right-hand side of this zone having considerable performance enhancement potential. This is problematic as some athletes may still be gaining an unfair performance advantage compared to others, even when operating within the “clean” zone.

As well as differences between athletes on the range of behaviors they adopt within the “clean” zone, athletes may themselves differ over time in what they view as acceptable clean sport behaviors. Whilst athletes—like all people—are motivated to act in a way that allows them to maintain a positive self-view, it is possible to act in ways that violate one's moral standards and still achieve this as long as one can justify and/or rationalize the behavior (Bandura, 1991). This can be achieved through moral disengagement, which is a collective term for eight psychosocial mechanisms that people use to justify and rationalize behavior that violates their moral standards (Bandura, 1991). Thus, some athletes may start adopting behaviors situated within the “gray” zone whilst maintaining a positive self-view through moral disengagement. Moral disengagement is heavily dependent on contextual factors, and a situation like that described earlier whereby some athletes are gaining an unfair advantage through “clean” but ethically questionable behaviors is likely to facilitate moral disengagement and encourage adoption of such behaviors by a greater number of athletes. Over time, an athlete could therefore change markedly in his/her performance-enhancement practices whilst all the time defining themselves as a clean athlete. The potential of moral disengagement to facilitate this process is supported by research that has demonstrated its use to justify and rationalize prohibited performance enhancement practices and maintain a positive self-view (Boardley and Grix, 2014; Boardley et al., 2014, 2015, 2017). Further, life history accounts of recently retired high-performance athletes have shown that progression in performance enhancing substance use can be driven by situational factors (e.g., urgency for improved performance, coaches, peers; Smith and Stavros, 2020).

Such behavioral changes may be due to changes over time in an athlete's performance enhancement mindset, with athletes adjusting their perception of what constitutes clean sport to accord with their performance enhancement mindset (Petróczi et al., 2017, 2021). Thus, over time an athlete's application of the term “clean sport” may vary so whilst the term stays static, the behaviors that underpin it change considerably. This argument is consistent with contemporary theory and empirical evidence relating to the development of a performance enhancement mindset. Specifically, both the Incremental Model of Doping Behavior (IMDB; Petróczi, 2013) and the gateway hypothesis of doping in sport (Backhouse et al., 2013) suggest doping evolves from routine application of non-prohibited performance enhancement practices (e.g., nutritional supplement use for performance enhancement). Accordingly, qualitative research has provided accounts from athletes across a range of sports that describe a process in which athletes move from no substance use at all, to use of nutritional substances, before finally progressing to prohibited performance enhancement methods (Boardley and Grix, 2014; Boardley et al., 2014, 2015). This process likely occurs alongside changes in athletes' sport participation, motivational climate, and goals within sport. Specifically, as athletes progress from grassroots sport primarily for enjoyment to competitive sport for achievement purposes, their progression as an athlete becomes much more dependent on exogenous (i.e., non-prohibited or prohibited) means of performance enhancement (Petróczi, 2013). In line with these changes, the athlete's mindset likely becomes much more focused on performance enhancement. Whilst not all athletes will progress to doping, even progression to unethical practices that are within the rules is problematic from a fair play and spirit of sport perspective. Consistent with this proposed progression, a meta-analysis of risk factors for doping found having experience with using nutritional supplements for performance enhancement was one of the strongest predictors of doping behavior (Ntoumanis et al., 2014). However, personal values and morals from early life experiences that prioritize authenticity over superiority and process over outcome may be protective even for athletes exposed to external factors that encourage progression of one's performance enhancement mindset (Williams et al., 2020; Petróczi et al., 2021; Shelley et al., 2021).

## Implications for Anti-Doping Education

Based on the conclusions from the previous section, it is important that education focuses on the development of protective factors that may guard against external factors that facilitate progression toward the “gray” area and/or prohibited substance use. If we accept that doping is a wicked problem to a considerable degree, and that wicked problems are complex, dynamic, multi-faceted, and intractable, then it follows that anti-doping education cannot be based upon universal, singular guidance. Due to the very nature of the doping problem, anti-doping education cannot seek to offer answers for all possible scenarios, but instead should take a more pragmatic approach by adopting approaches that allow for situated solutions.

Sensemaking is one such approach, representing the cognitive processes through which people develop a cognitive map of their situation by embedding an event within a familiar framework with personal meaning for them (e.g., their value system; Weick, 1995). Through appropriate training people can develop this skill to increase their ability to assess accurately the situations they find themselves in and make appropriate decisions. Sensemaking is particularly useful in circumstances that are troubling, conflicting, uncertain and/or ill-defined which is likely the case for many athletes when confronted with situational constraints conducive with adopting prohibited performance enhancement methods. The potential utility of sensemaking in the context of clean sport and anti-doping is consistent with arguments that doping can only be understood as a situated activity (Hauw, 2013). However, it goes beyond mere interpretation of a situation, which only requires describing what a troubling situation means, by also reflecting on how the situation has been constructed. It is also about more than just decision making as it is primarily concerned with defining what a decision is about rather than just what the correct decision is. It can therefore help individuals develop context-specific reasons for action, which are crucial to effective formation of decisions, intentions, and therefore action (Westaby, 2005).

Based on the above arguments and the successful application of sensemaking within ethical decision making (Brock et al., 2008; Kligyte et al., 2008; Mumford et al., 2008; Harkrider et al., 2013; Johnson et al., 2014; Bagdasarov et al., 2015), we propose that sensemaking training could be a successful addition to anti-doping education. Regardless of whether the motives underpinning intentional doping are immoral (e.g., trying to gain an advantage over the opposition) or otherwise (e.g., performance or aesthetic enhancement), the perpetrator is knowingly breaking the rules and therefore is committing an unethical act. Sensemaking training could be used to train athletes to be more aware of their thought processes, increase their awareness of their automatic judgments, and encourage them to fully analyze a problem and define what the decision is actually about prior to making a decision. Taking an example of a potential risk situation for doping, an athlete may be encouraged to dope by a coach when no longer progressing in their sport despite high levels of effort. Following sensemaking training, an athlete in this situation should be able to more thoroughly consider the meaning of their thought processes (e.g., recognize that they would likely make a different decision if they take personal responsibility for the act rather than displacing responsibility to the coach), be aware that the high value they place on developing their athletic ability can lead to automatic judgments that favor performance enhancement, and define that the decision is also about *how* they progress as an athlete and not just *whether* they progress.

Sensemaking approaches to anti-doping education should address both the reasons for and against doping, as the reasons for and against a behavior are not necessarily polar opposites (Richetin et al., 2011, 2012, 2020). Reasons for and against doping likely depend on separate goals and thought processes and as such both should be addressed independently rather than assuming merely reversing reasons for doping provides us with protective factors against it. This asserts the need to develop education programmes that aim to prevent doping and promote clean sport behavior separately. This contrasts with existing approaches that often tacitly assume homogeneity in reasons for doping. It is also consistent with recent evidence for significant idiosyncrasy in decisions about doping (e.g., Woolf and Mazanov, 2017) and clean sport (Petróczi et al., 2021).

Given the role of intuitive evaluations in decision making and the influence of values on such evaluations (Sonenshein, 2007), values-based education is a central component of the sensemaking process, although this is a two-way process. On the one hand, effective sensemaking training in anti-doping would help athletes elucidate their understanding of the value they place on how they enhance performance and establish clear boundaries for decision making when facing situations in which they are potentially vulnerable to doping. This could be helped by making anti-doping value frames stronger and more central and providing athletes with the skills to confront difficult situations without doping whilst also staying true to their athletic identity and the associated value they place on sporting performance. On the other hand, values-based anti-doping education must shift away from a set of abstract values such “respect,” “excellence,” “friendship,” “equality,” “courage,” and “fairness,” to acknowledging the existence of external value differences (e.g., Mazanov et al., 2019; Woolway et al., 2021) and internal value priorities (e.g., Mortimer et al., 2021) and working with them.

## Connecting Anti-Doping Education to Individual Decision Making

The ISE (WADA, 2021a) specifies four major components that should be part of all education plans. One of these—values-based education—is not doping-specific as it focuses more on the development of a strong moral basis for integrity and rule-following. The other three components are doping-specific, and consist of awareness raising, information provision, and anti-doping education (i.e., WADA code compliance). The guidelines for ISE implementation map these four components onto the athlete development pathway to illustrate the point/s at which athletes should receive education delivery relevant to each component (WADA, 2020).

It is assumed that this mapping of the four components onto the athlete development pathway is done to optimize the impact of education on athlete decision making regarding doping. By connecting education components to the process of individual decision-making, we can: (a) maximize the potential of athletes to make desirable choices about performance enhancement through relevant and situated education, and (b) provide conceptual clarity of how different components exert influence on decision making, which is paramount for devising meaningful outcome-based evaluation.^2^ This proposed link between mapping of education delivery and athlete decision making is explicit in the guidelines for two of the four components (WADA, 2020 p. 44, Table 5.2). First, it is stated that values-based education “builds the participant's capacity to make decisions to behave ethically.” Second, anti-doping education is proposed to help “build competence in clean sport behaviors and make informed decisions.” For values-based education, the guidelines for the ISE describe an “upstreaming” strategy whereby this form of education should be mainly focused during the early stages, with follow-up reinforcements at later stages to encourage continued development around ethical decision making. To complement this, anti-doping education focused on code compliance is targeted at a limited pool of athletes consistent with a “down streaming” strategy. This form of education must cover the mandatory topics set out in the WADA Code Article 18.2, such as principles and values associated with clean sport, the principle of strict liability and the consequences of doping (WADA, 2021b).

**Figure 2:**
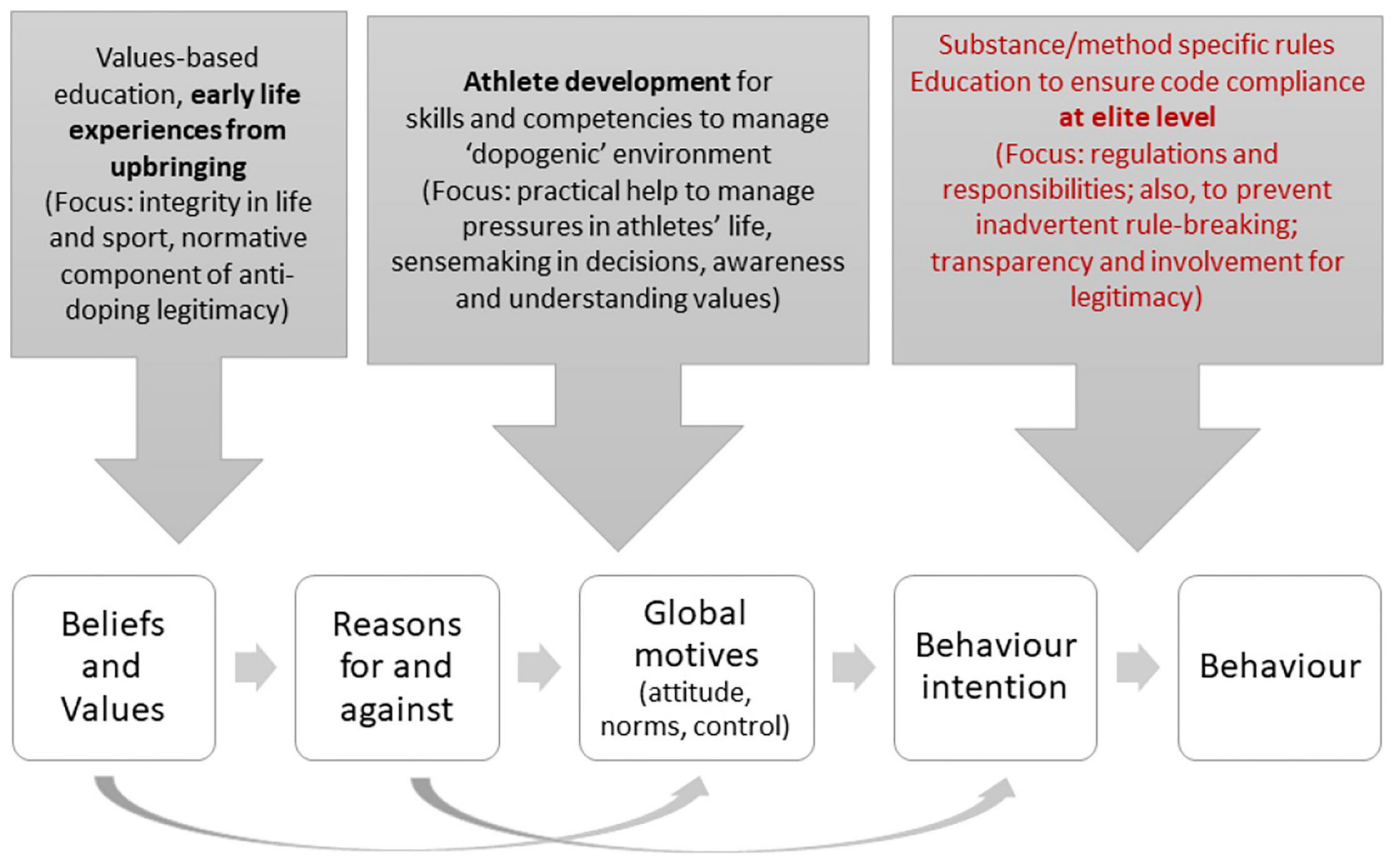
Mapping educational components to decision making about doping.

Whilst the link between two components of education delivery and athlete decision making is explicitly made in the guidelines, how this is expected to happen is not considered. To address this omission, here we set different aspects of education against key aspects of the decision-making process, juxtaposed on stages of Westaby's (2005) Behavioral Reasoning model (see Figure 2). First, values-based education is thought to develop personal integrity by influencing general values and beliefs about fairness, rule-following, and authenticity, and is largely rooted in early life experiences and upbringing and not specific to doping (Petróczi et al., 2021). For athletes who define clean sport in rule-based terms, values-based education may be of greatest relevance. Values-based education seeks to develop athlete integrity and athletes' ability to make the right choices about enhancing performance. Based on the available evidence (Overbye et al., 2013; Williams et al., 2020) and previous theories about motives for doing something and not doing it (Westaby, 2005; Richetin et al., 2011), it is fair to assume that values relating to sport integrity are necessary and sufficient to ensure clean sport behavior; but the lack of them is only necessary but not sufficient for doping use. The latter is always driven by some tangible, outcome-focused reason (Kirby et al., 2011; Overbye et al., 2013; Engelberg et al., 2015), for which values are adjusted to avoid or temper internal conflicts. Furthermore, values-based education is not merely telling athletes about the values of sport, it is about trying to instill such values in athletes. As such, values-based education should start early in athletes' careers, well before they are aware of prohibited substances and anti-doping (i.e., children and school sport athletes). To maximize the effectiveness of such education, a concerted effort is required whereby all significant others (e.g., parents, coaches, teachers) in an athlete's life reinforce this approach such that ethical values are deeply embedded in the broader environment (e.g., school curricula, home environment).

Next, we have the development of athletes' skills and competencies to manage situations in which they may be most vulnerable to doping focused on coping mechanisms, sensemaking, and awareness and understanding of person values underpins the core values and beliefs that influence global motives (e.g., attitudes; perceptions of social norms; personal control). The focus here should be on developing life skills during education that help children make decisions in the right way and cope with stress and pressure without resorting to unethical means. This should be targeted at athletes in the development pathway, but before they have reached international level (i.e., youth and talented athletes).

Finally, focused anti-doping education consisting of awareness of doping, accurate information on prohibited substances/methods and risks for inadvertent doping (e.g., supplements, unauthorized medication use) have important influences on behavior-intention formation and progression from intention to execution. Such education should center on code compliance and be aimed at elite athletes (i.e., national and international athletes). Athletes who define clean sport in substance-based terms may find compliance-based education of greatest relevance. Once athletes start thinking about performance enhancement in terms of “what they are allowed to do,” values-based education is likely to have limited impact. Key elements of compliance-based education are anti-doping rules and regulations, consequences of non-compliance, roles, and responsibilities, and how to prevent inadvertent doping. Compliance-based education is likely of greatest relevance to athletes who define clean sport in substance-based terms, providing them with education on what is required for code compliance regarding intentional doping, and what is required to minimize the risk of inadvertent doping. This form of education should offer practical advice and specific help to deal with known pressure points, recognizing performance enhancement is inherent in most athletes at this point (Petróczi et al., 2017).

## Caveats

This paper builds on the premise that doping represents the use of prohibited means and is therefore against the rules (WADA, 2021a). We took the position that if doping is against the set of rules that athletes unilaterally subscribe to in order to participate in sport, then breaking such rules represents cheating in the sport context. Doping is therefore cheating as long there are rules in place prohibiting such practice. This narrow and situated concept of “doping as cheating” appears to be widely accepted in the anti-doping literature, mostly as tacit positioning through terminology (e.g., “cheating in sport,” “drug cheats,” “cheaters”) but in some instances as reasoned argument (e.g., Schermer, 2008; Kirkwood, 2020), pushing the arguments toward challenging whether there is a sufficient ground for legitimising such rules. We further assumed that “doping is wrong” solely because such a proposition is at the core of preventive anti-doping education. Through the paper we do not intend to argue, or counterargue, whether doping should be allowed in sport. Nor do we question or support whether—once doping has been prescribed by the rules of sport—one should treat athletes as “cheaters” if they dope. We leave this argument to scholars in the fields of philosophy, ethics, and political science.

## Recommendations for Education

Based upon our arguments and observations to this point, we now propose some general recommendations for the delivery of education. First, informed by our model mapping educational components to decision making about doping (see Figure 2), values-based education should occur in schools and youth sport so that it occurs early in the athlete-development process, and much earlier than it does at present. Moreover, values-based education should be delivered by those responsible for athlete development rather than by education teams from Anti-Doping Organizations (ADOs). In contrast, doping-specific elements of education (i.e., awareness raising, information provision, and anti-doping education) should be delivered to athletes already involved in high-level competition when they are in or close to entering testing pools. Unlike values-based education, these aspects of the ISE should be delivered by education teams from organizations with responsibility for anti-doping educations, such as international and national sport federations, and regional and national anti-doping organizations (WADA, 2021a).

Second, developers of anti-doping education programmes must be clear on the goals they want to achieve through their programmes. The components of the programme should then be aligned with these specific objectives. Once specific objectives and programme components are aligned, it is then possible to develop an unequivocal strategy for evaluating the education programme. Monitoring and evaluation of education programmes is a key priority, as currently there is very limited knowledge regarding the effectiveness of anti-doping interventions and education programmes, including their development, implementation, and long-term evaluation (Boardley et al., 2021). When evaluation has taken place, interpretation has been hindered significantly by use of a miscellaneous soup of social-cognitive measures with no clear idea of what constructs were targeted by the intervention in the first place.

Third, we need to consider changing the language around anti-doping and start talking about protecting the *integrity* of sport, rather than *clean* sport. As we have discussed earlier, the term “clean” has multiple meanings amongst athletes, and as such current use of this term likely leads to disparate interpretations across athletes. Also, clean sport has to date been very closely aligned with anti-doping, with some people—incorrectly in our opinion—going as far as defining it as doping avoidance (i.e., the active non-use of doping substances and methods when competing in sport; Mortimer et al., 2021). As discussed earlier, being a clean athlete is so much more than merely not doping. By shifting the focus toward protecting the integrity of sport, we move away from this doping-centric focus and move toward a focus on the promotion of high levels of integrity more broadly. Whilst this change is yet to happen at the highest levels of the anti-doping system (i.e., WADA), we are starting to see this reflected in other areas of sport governance through the creation of integrity units by international sports federations (e.g., Athletics Integrity Unit; https://www.athleticsintegrity.org/) and ADOs (e.g., Sport Integrity Australia; https://www.sportintegrity.gov.au/what-we-do/anti-doping). This change would hopefully help people view doping as one of the many infringements against the integrity of sport, which in turn should promote the conceptualization of doping in terms of rule-breaking more generally.

## Conclusion

Wicked problems, such as doping, cannot be solved but they can be tamed. Conventional solutions not only fail to tackle them but may even exacerbate the issue by inadvertently making it even more complex. To reverse this paradoxical situation, we argue for critical and constructive analysis of relevant regulatory, normative, and cognitive systems to maximize legitimacy of the anti-doping system and address current misalignments between goals, strategies, and assessments. How we define clean sport should be an important aspect of this analysis, as continued reference to “clean sport” as doping-free sport hinders the development of the field and increases the already significant gap between prohibited and non-prohibited performance-enhancing practices and measures in place to protect clean sport. The former is characterized by rapid developments in pharmacology (e.g., carefully calibrated micro dosing, combination of microdosing and dietary supplements) and technology to boost performance and training methods (e.g., continuous glucose monitors). The anti-doping movement needs to recognize this and set goals and strategies for research, testing, and education that are better aligned with this fast-paced development in performance enhancement techniques. Regarding education, we need to better match the adopted approaches to individual decision making and develop athletes' sensemaking skills to better prepare them for the uncertain situations they are likely to find themselves in with respect to doping. Through the adoption of the recommendations proposed here, we believe it is possible to see real progress in the promotion of clean sport over the coming years.

## Author Contributions

Both authors listed have made a substantial, direct, and intellectual contribution to the work and approved it for publication. The authors contributed equally to the manuscript.

## Conflict of Interest

The authors declare that the research was conducted in the absence of any commercial or financial relationships that could be construed as a potential conflict of interest.

## Publisher's Note

All claims expressed in this article are solely those of the authors and do not necessarily represent those of their affiliated organizations, or those of the publisher, the editors and the reviewers. Any product that may be evaluated in this article, or claim that may be made by its manufacturer, is not guaranteed or endorsed by the publisher.

## Footnotes

^1^An earlier version of the model was presented in an Invited Keynote lecture at the Second WADA Global Education Conference, Beijing, China, October 2018 (Petróczi, A.: “Research on athletes' perspectives on clean sport”).

^2^Outcome-based evaluation assesses the difference the intervention made and focuses on the impact of the intervention (e.g., better understanding, increased knowledge, competency, desirable behaviour choices, agency at individual level; and reduction of AAFs and ADRVs at population level). Output-based evaluation concerns with reach and intensity and assessed via the activities (frequency of intervention, athletes reached, events attended, information leaflets distributed, number of visitors in the outreach booths, etc.).
